# The Motor Competence of Learning with a Perception-Awareness Approach: A Pilot Study in the Formal Learning Environment

**DOI:** 10.3390/children13081019

**Published:** 2026-07-31

**Authors:** Rosario Ceruso, Giuseppe Giardullo, Manuele Taleb, Giuseppe Di Lascio, Gaetano Raiola

**Affiliations:** 1 Research Center of Physical Education and Exercise, University of Pegaso, 80143 Naples, Italy; rosario.ceruso@univr.it (R.C.); giuseppe.giardullo@univr.it (G.G.); giuseppe.dilascio@univr.it (G.D.L.); gaetano.raiola@unipegaso.it (G.R.); 2 Department of Neuroscience, Biomedicine and Movement, University of Verona, 37129 Verona, Italy

**Keywords:** physical education, motor skills, motivation, primary school

## Abstract

**Background/Objectives:** Physical activity in primary school is increasingly recognized not only for developing skill-related fitness, but also for promoting well-being, motivation, and awareness of individual abilities. However, studies integrating quantitative performance data with qualitative perceptual data in school settings remain limited. This study aimed to explore the associations between objectively measured motor competence and students’ perceptions of enjoyment, motivation, and perceived competence during primary school physical education. **Methods**: This observational pilot study involved 17 students (mean age: 10.6 ± 0.5 years). The quantitative assessment included the Yo-Yo Test for intermittent endurance and the Sargent Test for lower-limb explosive strength, along with anthropometric measurements (mean height: 1.44 ± 0.09 m; mean weight: 39.9 ± 6.3 kg). A dichotomous (yes/no) questionnaire assessed enjoyment, motivation, perceived competence, and interest in physical activity. Data were analyzed using descriptive statistics, the Shapiro–Wilk test, Pearson’s correlation, and the Chi-square test. **Results**: Significant correlations emerged between height and body weight (r = 0.667; *p* = 0.003) and between body weight and Sargent Test performance (r = 0.544; *p* = 0.024). Biological sex was also significantly correlated with body weight (r = −0.562; *p* = 0.019). The Chi-square analysis showed significant associations between motivation and positive perception of physical activity (*p* < 0.05), particularly regarding sports participation and peer-related engagement. **Conclusions**: Despite differences in motor performance, students’ perceptions of physical activity were generally positive. Anthropometric characteristics influenced performance, while motivational and social factors were strongly associated with participation. Integrating performance and perceptual data provides a more comprehensive understanding of psychophysical development during school age.

## 1. Introduction

In recent years, physical activity and sport in schools have progressively been recognized as a fundamental component of the educational process, not only for its physical benefits, but also for its contribution to the cognitive, emotional and social development of students [1]. In primary school in particular, physical education is one of the first structured contexts in which children experience movement as a shared, regulated and learning-oriented experience, laying the foundations for their future relationship with physical activity and sport [2]. Numerous studies have shown that physical experiences during childhood are associated not only with the development of basic motor skills, but also with the construction of attitudes, beliefs and motivational dispositions that can influence adherence to a physically active lifestyle during adolescence and adulthood [3,4]. Traditionally, the assessment of physical activity in schools has focused mainly on performance or functional indicators, with particular attention to coordination and technical skills in children [5]. However, a growing body of evidence suggests that this approach is partial, especially in younger age groups, where participation and commitment are strongly influenced by the subjective experience of physical activity [6]. In this respect, how children perceive physical activity by children, in terms of pleasure, satisfaction, competence, and peer relationships, can have a significant impact on adherence, commitment, and future predisposition towards sports practice [7,8].

Within this framework, Self-Determination Theory (SDT) is one of the most widely used theoretical models for interpreting motivational processes in education and motor skills. According to this perspective, the quality of the learning experience depends largely on the satisfaction of three basic psychological needs: competence, autonomy and relatedness. When these needs are supported, individuals tend to develop more self-determined motivation, associated with greater involvement, enjoyment and persistence over time; conversely, contexts that frustrate these needs can generate negative experiences, disaffection and early dropout [9,10]. In the context of school physical education, numerous qualitative and quantitative studies have shown that the motivational climate created by the teacher and the way activities are organized significantly influence students’ enjoyment, participation and well-being [11,12,13].

A particularly relevant aspect concerns the relationship between actual physical competence and perceived competence. Theoretical models hypothesize that, during development, the perception of competence may mediate the relationship between motor skills and participation in physical activity, favoring positive or negative trajectories of involvement [14]. However, more recent evidence suggests that this relationship is neither linear nor immediate, especially in primary school age children, where enjoyment and pleasure in the activity can remain high even in the presence of heterogeneous performance levels [15].

Examining actual motor competence together with perceived competence is relevant because these two dimensions, although theoretically related, do not necessarily develop in parallel. Objective motor performance describes what a child is able to do under standardized assessment conditions, whereas perceived competence reflects how the child interprets and evaluates his or her own abilities. A discrepancy between these dimensions may have important educational implications. Children with relatively low motor performance may still report positive perceptions and high motivation, while children with adequate motor competence may underestimate their abilities and show lower willingness to participate. Consequently, assessing only objective performance or only subjective perception may provide an incomplete representation of the child’s motor experience. An integrated assessment may therefore help clarify whether children’s perceptions are consistent with their measured abilities and whether motivational and social responses are associated with actual performance within the formal physical education setting.

In youth football, a difference has also been observed between objective technical assessment and qualitative perception of performance [16].

At the same time, the contemporary literature on physical education emphasizes the importance of pedagogical approaches that value physical experience in its entirety, including emotional, social and motivational dimensions [17]. Concepts such as physical literacy and meaningful physical education emphasize the building of confidence, motivation and a sense of meaning through movement, proposing a view of physical education as a multidimensional educational experience [18]. From this perspective, enjoyment and perceived competence are not just secondary outcomes, but key indicators of the quality of the educational intervention [19].

Despite this growing theoretical recognition, evidence from formal primary school physical education remains fragmented. Most studies assess either motor performance or motivational and perceptual outcomes, while fewer investigations examine whether objectively measured competence corresponds to children’s perceptions of their own abilities within the same educational context. This distinction is important because motor performance and perceived competence may show different patterns of association with enjoyment, motivation, and participation. Many studies focus exclusively on physiological aspects or, conversely, exclusively on motivational questionnaires, without any real dialog between the two dimensions.

Previous studies have investigated motor competence, perceived competence, and motivation in children from different perspectives, often focusing on a single dimension or examining these variables in intervention-based or sport-specific contexts. Other investigations have explored associations between motor competence and psychosocial outcomes, but relatively few have simultaneously combined objective motor testing with students’ subjective perceptions within routine primary school physical education lessons. Furthermore, most available evidence has primarily emphasized performance outcomes or motivational constructs separately, providing limited information on the extent to which objectively measured motor competence corresponds with children’s own perceptions in everyday educational settings. Therefore, the present pilot study contributes preliminary evidence by integrating objective motor assessments and perceptual measures within the same school-based observational framework. An additional unanswered question concerns whether children’s subjective perceptions of their own motor competence accurately reflect their objectively measured performance during routine physical education lessons. This issue is particularly relevant in formal primary school settings, where physical education is designed not only to develop motor competence but also to promote enjoyment, motivation, and positive attitudes toward lifelong physical activity. Understanding whether objective performance and perceived competence converge or diverge in this educational context may help teachers better interpret students’ engagement and adapt teaching strategies to support both physical and psychosocial development.

### Purpose of Study

The aim of this pilot observational study was to explore the relationships between objectively assessed motor competence and children’s perceived competence, enjoyment, and motivation toward physical activity within the context of formal primary school physical education. A secondary aim was to examine whether objective motor performance corresponded with students’ subjective perceptions of their own abilities.

## 2. Materials and Methods

### 2.1. Study Participants

This study employed an observational cross-sectional pilot design aimed at integrating objective measures of motor competence with students’ subjective perceptions of physical activity within the formal primary school setting. Data collection was conducted during routine physical education lessons without any intervention, manipulation of curricular activities, or modification of the educational program. The study was designed to provide an exploratory description of motor performance and perceptual variables and to examine potential associations between these dimensions. The study involved 17 primary school children (8 boys and 9 girls) attending a fifth-grade class, with a mean age of 10.6 ± 0.5 years. Eligibility was verified through information provided by parents and school records. Children with known medical conditions, musculoskeletal injuries, or other health issues limiting participation in regular physical education were not included in the study. Although limited, the sample size was considered adequate for the purposes of the study, which is a descriptive and exploratory pilot study aimed at providing an initial integrated analysis of objective performance indicators and subjective perceptions of physical experience in schools.

### 2.2. Study Design

The study was structured according to an observational, cross-sectional, quantitative–qualitative design with qualitative and quantitative methods, with the aim of integrating the assessment of motor performance with the analysis of students’ subjective perceptions of school physical activity. Data collection took place within the normal school context and involved a single evaluation phase, without experimental interventions or manipulation of curricular activities. The quantitative component concerned the assessment of specific motor skills considered relevant at developmental age. In particular, the ability to sustain intermittent effort was assessed using the Yo-Yo Test, with the number of completed shuttles as the main indicator, while the explosive strength of the lower limbs was measured using the Sargent Test, performed in three consecutive tests in order to obtain a reliable estimate of jump height.

Three Sargent Test trials (hS1, hS2, hS3) were recorded, and each trial was analyzed separately to examine potential differences across repeated attempts.

Baseline anthropometric measurements were taken to describe the sample and provide interpretative context to the performance data. In parallel, a qualitative-quantitative assessment of students’ perceptions was conducted through a structured questionnaire with a dichotomous response (yes/no). The questionnaire was designed to investigate enjoyment of physical activity, motivation to practice, perception of competence with respect to one’s motor skills, and interest in the social and improvement dimension of sports activity. The integration between quantitative performance tools and subjective perceptions responds to recent methodological orientations in motor sciences [20]. It was administered in a family context for the students, with the aim of promoting spontaneous and understandable responses to the age group considered divorced from the competitive context to avoid the halo and assimilation effects. The questionnaire with quantitative variables can be observed in the following Table 1.

The questionnaire was specifically developed for the purposes of this pilot study and was intentionally designed using dichotomous (Yes/No) responses to ensure simplicity, comprehensibility, and ease of administration for primary school children. Given the exploratory nature of the study and the young age of the participants, a brief response format was considered appropriate to minimize respondent burden and facilitate consistent understanding of the questions. Before administration, the questionnaire was reviewed by primary school teachers to ensure that the wording was age-appropriate and easily understandable. The questionnaire was completed individually by the children in a calm and familiar home environment, outside the competitive school setting, to minimize social desirability and contextual pressure. Parents were instructed only to clarify the wording of the questions when necessary and not to influence the children’s responses. Parents were instructed to provide assistance only when necessary to clarify the wording of the questions and not to influence the children’s responses. Questionnaire responses were coded as Yes = 1 and No = 0 prior to statistical analysis. Because the questionnaire was specifically developed for this exploratory pilot study, no formal psychometric validation (e.g., internal consistency or construct validity) was performed. Each item was analyzed individually, and no composite scores were calculated, as the questionnaire was intended to provide preliminary descriptive information rather than measure latent psychological constructs.

### 2.3. Statistical Analysis

Statistical analysis was performed using the JASP software 0.95.0.0. Given the size of the sample and the observational nature of the study, the analysis was mainly based on a descriptive and exploratory plan. For quantitative variables, mean and standard deviation were calculated, and the normality of the distributions was verified using the Shapiro–Wilk test.

Pearson’s correlation coefficient was used to explore linear associations between quantitative variables. Although the Yo-Yo Test scores showed a significant deviation from normality, the analysis was retained because the study was designed as an exploratory pilot investigation and the correlations were intended to provide preliminary indications rather than confirmatory evidence. Given the limited sample size and the non-normal distribution of the Yo-Yo Test scores, the corresponding results were interpreted with caution and were not used to support definitive conclusions.

Because the primary objective of the study was exploratory, Pearson correlation coefficients were used to investigate linear associations between quantitative variables. Given the pilot nature of the study and the limited sample size and the number of statistical comparisons performed, the results should be interpreted with caution. The possibility of type I errors cannot be excluded, and the observed associations require confirmation in larger and adequately powered studies.

The questionnaire responses were analyzed by calculating the frequencies and percentages of affirmative and negative responses for each item, as well as by descriptive statistics of the coded responses. The relationships between quantitative variables were explored using Pearson correlation in order to identify any linear associations between anthropometric variables and performance in motor tests. Furthermore, the associations between qualitative variables of the questionnaire were verified through the Chi-square test, in order to identify any statistically significant relationships between the different perceptual and behavioral dimensions related to sports practice. With the aim of exploring the connection between the performance and perceptual dimensions, the variables related to the motor tests (Yo-Yo Test and Sargent Test) were related to the appropriately coded questionnaire responses, analyzing any associations between levels of performance and perception of physical practice, motivation, and appreciation of physical activity.

### 2.4. Ethics Committee

This research was conducted in accordance with the ethical principles established for educational and social studies involving minors. The study involved the collection of anthropometric data (height and weight), the administration of standardized motor tests (Sargent Test and Yo-Yo Test), and the completion of anonymous questionnaires aimed at investigating students’ perceptions of enjoyment, motivation, and perceived competence related to physical activity. No experimental interventions or manipulation of variables were performed. All procedures were conducted in accordance with the ethical principles outlined in the Declaration of Helsinki and its subsequent revisions, ensuring respect for the dignity, autonomy, and confidentiality of the participants. Prior to data collection, clear information about the aims of the study, the procedures involved, and the voluntary nature of participation was provided. Participation was entirely voluntary, and data were collected and analyzed anonymously. The study was conducted in accordance with internationally recognized ethical standards. The research was designed and conducted by the Research Centre Physical Education and Exercise of Pegaso Digital University of Naples, and all procedures complied with the ethical principles established by the Centre’s Ethics Committee under protocol code PROT/E 004726 dated 15 July 2025.

## 3. Results

The descriptive analysis of quantitative variables was conducted on a sample of 17 students (8 boys and 9 girls). Anthropometric measurements highlighted an overall homogeneous profile consistent with the school age of the sample: the mean height was 1.442 m (SD = 0.085), with values ranging from 1.300 to 1.580 m, while body weight showed a mean of 39.924 kg (SD = 6.341), with a range between 30.0 and 51.0 kg. In both anthropometric variables, the Shapiro–Wilk test showed no significant deviations from normality (height: *p* = 0.609; weight: *p* = 0.693), suggesting a substantially regular distribution of values within the group. Results are summarized in Table 2.

Regarding the intermittent resistance component assessed through the Yo-Yo Test, the number of shuttles completed (shuttles) recorded an average of 4.235 (SD = 1.033), with values ranging from 3 to 6. Unlike anthropometric measures, the distribution of scores on the Yo-Yo Test did not meet the normality criterion (Shapiro–Wilk: *p* = 0.028), indicating greater heterogeneity in the ability to sustain intermittent effort among students. Interindividual differences in intermittent capacity in developmental age are frequently associated with different levels of sports practice and biological maturation [21,22]. This trend suggests the possible presence of performance subgroups, highlighting a more marked functional variability than that observed in anthropometric characteristics. Evaluation of lower limb explosive strength, measured by the Sargent Test on three consecutive trials (hS1, hS2, hS3), showed progressively higher mean values along the repetitions. In particular, the jump height at the first test (hS1) was found to be 21,588 cm (SD = 5.328), with values ranging from 11 to 30 cm; at the second test (hS2) the mean increased to 24.529 cm (SD = 4.274), with a range between 17 and 32 cm; finally, at the third trial (hS3) the mean value reached 25,941 cm (SD = 3.766), with values between 18 and 32 cm. In all three trials, the Shapiro–Wilk test showed no significant deviations from the normal distribution (hS1: *p* = 0.446; hS2: *p* = 0.685; hS3: *p* = 0.323), indicating a substantially regular distribution of jumping performance in the sample. The increasing trend in mean values between hS1, hS2, and hS3 also suggests a possible familiarization effect with the gesture or a progressive improvement in executive effectiveness between trials, rather than a change attributable to training interventions (given the nature of the measurement in repeated trials in the same context). Similar trends have been observed in previous studies of young athletes, where close repetitions result in immediate executive improvement. Overall, while the Sargent Test showed a relatively orderly and consistent distribution of performance, the Yo-Yo Test showed a greater dispersion of results, outlining a profile in which the jumping ability appears more uniform than the ability to sustain intermittent effort. Pearson Correlation was then applied to highlight any correlations between the variables related to the applied tests. The results are reported in Table 3.

Pearson’s correlation coefficient results highlight different relationships between performance on the Yo-Yo Test (Shuttles) and jump measures on the Sargent Test, as well as between the three Sargent Test detections. In particular, the correlation between Yo-Yo Test (Shuttles) and Sargent Test hS1 is negative, very weak (r = −0.106) and not statistically significant (*p* = 0.685). Similarly, the relationship between Yo-Yo Test (Shuttles) and Sargent Test hS2 shows a very weak (r = 0.069) and non-significant (*p* = 0.792) positive correlation. The correlation between Yo-Yo Test (Shuttles) and Sargent Test hS3 also appears negative, very weak (r = −0.093) and not significant (*p* = 0.724). Overall, these results indicate that performance in the Yo-Yo test is not significantly associated with vertical jumping ability measured in the three Sargent Test trials, suggesting that intermittent aerobic capacity and vertical explosive strength represent relatively independent performance components in the analyzed sample, while additional relationships between anthropometric variables, biological sex, and performance measures are reported in Table 4.

A significant negative correlation emerged between biological sex and body weight (r = −0.562; *p* = 0.019), indicating that weight distribution differed significantly between biological sexes in the analyzed sample. Similarly, significant negative correlations between biological sex and performance were observed in the Sargent Test, both in the hS1 test (r = −0.554; *p* = 0.021) and in the hS3 test (r = −0.499; *p* = 0.041). These results suggest that vertical jump performance varies significantly by biological sex, highlighting differences in explosive strength abilities between the groups analyzed. Regarding anthropometric variables, a significant positive correlation was observed between height and body weight (r = 0.667; *p* = 0.003), indicating that as height increases, students’ weight also increases. Furthermore, a significant positive correlation was found between body weight and performance in the Sargent Test hS3 (r = 0.544; *p* = 0.024). A positive association between body weight and jump performance was observed in the present sample. However, given the observational nature of the study and the limited sample size, this relationship should be interpreted with caution, as it may partly reflect differences in biological sex distribution and biological maturation rather than an independent effect of body weight. Overall, the results highlight how anthropometric characteristics and biological sex significantly influence some explosive force performance, highlighting the existence of statistically significant relationships between body structure and motor performance. Subsequently, the analysis of qualitative perceptual aspects was conducted, obtained by administering the questionnaire and the application of the Chi-square test. First, you can see the results in Table 5. Table 5 combines descriptive response frequencies by biological sex with the results of Chi-square analyses performed between selected pairs of questionnaire items. The frequencies are presented only to describe the distribution of responses within the sample, whereas the reported *p*-values refer exclusively to the associations between the questionnaire items indicated in the “Related questions” column and not to comparisons between boys and girls.

The analysis of associations between the questionnaire variables was conducted using the Chi-squared test, in order to verify any relationship between some students’ perceptions and behaviors regarding sports practice. The results show several statistically significant associations between some questions in the questionnaire. In particular, a significant relationship emerges between the question “Do you play sports in the afternoon?” and “Would you like to train more with your teammates?” (*p* = 0.026). This finding suggests that participation in afternoon sports activities is associated with the desire to train more with teammates, indicating how extracurricular sports practice can foster greater motivation for collective participation. A highly significant association was observed between the question “Are you satisfied with how high you jump and how fast you run?” and “Would you like to train more with your teammates?” (*p* < 0.001). This data suggests that the perception of one’s motor performance, in terms of jumping ability and running speed, is closely linked to the desire to increase training opportunities with teammates, highlighting a possible link between self-perception of motor skills and training motivation. Similarly, a significant relationship was found between “Would you like to jump higher and run faster?” and “Would you like to train more with your teammates?” (*p* = 0.001). This finding indicates that the desire to improve one’s motor skills is associated with the desire to increase opportunities for shared training, suggesting that motivation to improve performance can be supported by the social and collaborative context of sports activity. A further significant association emerged between “Would you like to play volleyball, basketball and football with your classes?” and “Would you like to train more with your teammates?” (*p* = 0.003). This finding highlights how interest in sports activities practiced with classmates is linked to the desire to increase training time with peers, highlighting the role of the social dimension of sport in promoting student engagement. Finally, a significant relationship was observed between “Do you play sports in the afternoon?” and “Do you enjoy participating in sporting competitions?” (*p* = 0.005). This finding suggests that students who participate in afternoon sports also tend to show a greater propensity to participate in sports competitions, highlighting how regular sports practice may be associated with a more positive attitude towards competitive activities.

### Connections of the Different Parameters

The *p*-values obtained in the different statistical analyses allow us to understand the nature of the relationships between the variables considered in the sample. First, the Shapiro–Wilk test allowed us to verify the distribution of quantitative variables. The results showed a normal distribution for anthropometric variables and Sargent Test measures (*p* > 0.05), while the Yo-Yo Test showed a significant deviation from normality (*p* = 0.028). This difference suggests greater heterogeneity in students’ intermittent endurance ability with respect to anthropometric characteristics and vertical jumping ability. Based on these distributional characteristics, the relationship between quantitative variables was subsequently analyzed using Pearson’s correlation. The results showed *p*-values consistently above the significance level (*p* > 0.05) for the relationships between performance in the Yo-Yo Test and performance in the Sargent Test, indicating the absence of statistically significant associations between intermittent resistance and vertical explosive force in the analyzed sample. This suggests that these two components of physical performance represent relatively independent dimensions in students’ motor development. Conversely, the analysis of qualitative variables using the Chi-square test highlighted several statistically significant associations between some questionnaire responses (*p* < 0.05). In particular, significant relationships emerged between afternoon sports practice, the desire to train with teammates, and the perception of one’s motor skills. These integrated relationships between quantitative and qualitative variables are presented in Table 6.

The association between the Yo-Yo Test and the Sargent Test refers to the overall pattern observed across the three Sargent Test trials (hS1, hS2, and hS3), all of which yielded non-significant correlations.

These results indicate that, while physical variables measured through motor tests do not show significant relationships with each other, perceptual and motivational variables related to sports practice are strongly interconnected. Consequently, the integrated reading of *p*-values highlights how the physiological dimensions of motor performance and the motivational and behavioral ones follow different dynamics: the former show limited statistical relationships in the analyzed sample, while the latter highlight significant associations that underline the role of the social and motivational dimension in participation in sports activities.

## 4. Discussion

The study aimed to analyze the impact of school physical activity in primary school in terms of liking, motivation, and perception of competence, integrating performance indicators (Yo-Yo and Sargent) and perceptual data (dichotomous questionnaire). The results converge in describing an overall very positive school motor experience, but also characterized by differentiated patterns between “pleasure in participating”, “desire to continue” and more sensitive dimensions such as perceived competence and social preferences. This finding is consistent with evidence showing that the motivational and performance dimensions can evolve partially independently [23,24]. The first two questions of the questionnaire show clear evidence: all students report enjoying physical activity and wanting to do it more often. This result, although descriptive, is very relevant because it indicates that physical education, at least in the observed context, produces a positive emotional and motivational experience that constitutes a key prerequisite for adherence and continuity of practice. School literature emphasizes that physical education experiences are not neutral: the way teachers structure learning activities and interact with students can substantially influence autonomy, competence, and relatedness, thereby shaping motivation and engagement. According to Self-Determination Theory, need-supportive teaching practices include providing meaningful choices during activities, adapting task difficulty to individual ability levels, offering constructive and competence-oriented feedback, encouraging cooperation among classmates, and emphasizing personal improvement rather than social comparison. Such strategies have been consistently associated with greater enjoyment, intrinsic motivation, and sustained participation in physical education [25]. It is important to note that motivation in primary school may be strongly driven by immediate pleasure, play, and relationships; however, the literature shows that structured, theory-driven (often SDT-based) school interventions can consolidate motivation, autonomy, and a sense of competence over time, making a shift toward extracurricular physical activity more likely [26]. When the focus shifts from “like” to “as I perceive myself”, more variability appears: a significant portion of students do not declare themselves fully satisfied with their ability to run and jump, while the desire to improve remains high. This pattern is consistent with the fact that perceived competence is a more delicate dimension: it depends not only on actual skills, but also on experiences of success/failure, feedback received, social comparisons, and opportunities to choose tasks with an adequate level of challenge. SDT interprets competence as a fundamental psychological need: when satisfied, it supports intrinsic motivation; when threatened, it can increase anxiety, avoidance, and disaffection [27]. The most variable item is that relating to the preference to play team sports (volleyball/basketball/football) with classmates. This data can be read as an indicator of the relatedness component: not all students experience social interaction in physical activities in the same way. Relational dynamics and the perception of inclusion represent central variables in determining physical involvement in developmental age [28]. In SDT, the quality of relationships and feeling accepted in the group are crucial for well-being and participation; experiences of exclusion or social confrontation can reduce pleasure even in the presence of well-designed physical activities.

In parallel, the analysis of the qualitative variables of the questionnaire through the Chi-square test allowed us to identify some statistically significant associations between the students’ responses. In particular, significant relationships emerged between extracurricular sports practice, the desire to train with classmates, and participation in sports activities. These findings suggest how the social and motivational dimensions of physical activity play a significant role in participation and interest in sports in schools. The presence of these associations highlights how motivation to participate in sports does not depend exclusively on individual motor skills but is also strongly influenced by the relational context and the opportunities for sharing physical activity with peers.

On the quantitative side, Yo-Yo shows a non-normal distribution, suggesting greater heterogeneity in intermittent endurance capacity. This is plausible in a school sample, where exposure to extracurricular sports, movement habits, fitness levels, and maturation may differ widely. Furthermore, the literature on Yo-Yo testing highlights the need to interpret results as a function of age, sex, and protocol (including variants “children” or age-adapted), and makes regulatory references available for youth populations, especially at ages 6–16 years [29]. In contrast, the Sargent (hS1–hS3) presents distributions compatible with normality and an increasing mean trend between trials, likely attributable to familiarization and optimization of the gesture.

Further investigation was conducted through Pearson’s correlation analysis, aimed at exploring the relationships between some anthropometric variables and motor performance. The results showed several statistically significant correlations. In particular, significant relationships emerged between biological sex and body weight, as well as between biological sex and performance on the Sargent Test, suggesting that biological sex -related differences may influence some components of explosive force in students. Furthermore, a significant positive correlation was observed between height and body weight, a result consistent with what was expected from a physiological and anthropometric point of view. A significant correlation was also found between body weight and vertical jump performance, suggesting that some morphological characteristics may influence the ability to express explosive force in the lower limbs. Overall, this evidence confirms the importance of considering anthropometric variables in the interpretation of motor performance in school settings.

Recent evidence from the sports field also confirms how different methodological approaches can produce specific adaptations on the functional level without automatically determining parallel variations in perceptual and emotional dimensions: for example, the comparison between traditional and ecological-dynamic approaches in Brazilian Jiu-Jitsu has shown differentiated effects on strength development, reactivity and perception of distress, highlighting the relative autonomy between performance adaptations and subjective experiences [30]. This perspective is consistent with the scientific evolution of Italian motor sciences, increasingly oriented towards an integration between educational, functional and social dimensions [31]. Reviews and meta-analyses of physical education interventions highlight effects not only on psychomotor outcomes but also on affective and social outcomes, supporting the importance of integrating objective and subjective indicators to assess the quality of experience [32]. From this perspective, recently proposed integrated assessment models for primary school teacher training underline the need to jointly consider physical, pedagogical and reflective dimensions, moving beyond exclusively performance approaches [33,34].

The integrated reading of the *p*-values obtained from the different statistical analyses allows a more complete view of the study results. Pearson correlations highlighted significant relationships mainly between anthropometric variables and motor performance, indicating how some physical characteristics can influence specific components of performance. Conversely, Chi-square analysis highlighted significant associations between variables related to perception, motivation, and sports participation. This difference suggests that biological and performance dimensions and perceptual and motivational dimensions follow partially distinct but complementary dynamics. The integration of statistical results shows that students’ physical development cannot be interpreted exclusively through performance indicators but requires a multidimensional reading that also includes motivational and behavioral aspects related to sports practice.

From a practical perspective, the present findings suggest that physical education teachers should adopt teaching strategies that combine objective assessment of motor competence with continuous attention to students’ subjective experiences. The coexistence of heterogeneous motor performance and consistently positive perceptions of physical activity indicates that enjoyment and motivation can be maintained even when skill levels differ among students. Therefore, differentiated activities, constructive feedback, opportunities for cooperative learning, and progressive task adaptation may help sustain students’ engagement while supporting individual motor development within inclusive physical education settings. These findings reinforce the importance of considering motivational, perceptual, and social dimensions alongside motor performance when planning and evaluating primary school physical education programs.

### Limits of the Study

Despite the interest of the results that emerged, this study has some limitations that must be considered when interpreting the data. First, the small sample size and the origin of a single primary school class limit the possibility of generalizing the results to other school contexts or to larger populations. The data obtained must therefore be interpreted as indicative and useful for generating hypotheses rather than as definitive evidence on the impact of school physical activity. However, this limitation is intrinsic to the exploratory nature of the study, whose main objective was to test the feasibility of an integrated approach between performance and perceptual measures.

The questionnaire did not include a validated multidimensional measure of perceived competence. Instead, perceived competence was explored through a simplified item assessing children’s satisfaction with their own motor performance. Although this approach was considered appropriate for an exploratory pilot study involving young children, future research should employ validated multidimensional instruments specifically designed to assess perceived motor competence.

In addition, one questionnaire item simultaneously addressed satisfaction with jumping and running performance. Although separating these constructs would have allowed a more specific assessment, the combined item was retained to maintain a brief and child-friendly questionnaire suitable for this exploratory pilot study. Future studies should consider evaluating these motor skills separately.

Furthermore, when analyzing the relationships between questionnaire items, multiple statistical tests were conducted, which increases the risk of Type I errors. In future studies, it is advisable to adjust for multiple comparisons.

The use of Pearson’s correlation for analyses involving the non-normally distributed Yo-Yo Test represents an additional methodological limitation. In combination with the small sample size, this may have reduced the stability of the estimated correlations. Therefore, these findings should be considered preliminary and should be verified in larger samples using non-parametric or robust statistical approaches.

A further limitation is represented by the observational and cross-sectional design, which does not allow us to establish causal relationships between school physical activity, motor performance and subjective perceptions. The lack of a longitudinal assessment prevents us from understanding whether and to what extent enjoyment of physical activity, motivation, or perceived competence can evolve over time in relation to continuous participation in physical activities. Future studies could benefit from longitudinal or quasi-experimental designs, capable of analyzing the effect of specific teaching approaches on the evolution of psychomotor and motivational variables.

## 5. Conclusions

The present findings indicate that positive perceptions of physical activity coexist with heterogeneous levels of motor performance in this sample. Although causal inferences cannot be drawn from this observational pilot study, the results support the value of jointly examining objective and subjective dimensions when investigating children’s experiences during physical education. The overall results outline a picture in which students’ motor experiences appear strongly positive in terms of pleasure and engagement, despite the presence of heterogeneous performance levels and subjective perceptions that do not always overlap with the motor skills detected. The analyses carried out highlighted how some anthropometric variables and individual characteristics are significantly associated with some motor performance, as emerged from Pearson’s correlations, suggesting that morphological aspects and individual differences may influence specific components of performance, particularly those related to explosive force. In parallel, the analysis of the questionnaire responses through the Chi-square test showed the presence of significant associations between some perceptual and behavioral dimensions of sports practice, highlighting the role of motivation, participation, and the social dimension of physical activity. In particular, the high appreciation of physical activity and the widespread desire to practice it more frequently suggest that school physical education represents a privileged context for promoting a positive relationship with movement already at an early age. This data takes on significant value when interpreted in light of the role that childhood motor experiences play in shaping future attitudes, motivations, and predispositions towards an active lifestyle. The study helps fill, albeit preliminarily, a gap highlighted in the literature by proposing an integrated approach to the assessment of physical activity in primary school. A joint reading of the statistical results suggests that the physical dimensions of performance and the perceptual and motivational dimensions follow partly different but complementary dynamics, highlighting the importance of jointly considering biological, experiential, and social aspects in the study of physical activity in developmental age.

The present findings indicate that positive perceptions of physical activity coexist with heterogeneous levels of motor performance in this sample. Although causal inferences cannot be drawn from this observational pilot study, the results support the value of jointly examining objective and subjective dimensions when investigating children’s experiences during physical education.

Future studies, with larger samples, longitudinal designs, and more sensitive assessment tools, should employ more differentiated self-assessment scales, validated multidimensional motivation questionnaires, and integrated assessment strategies combining objective motor testing with observational methods and self-report instruments.

## Figures and Tables

**Table 1:** Instruments used in the study.

Assessment Area	Instrument/Question	Variables/Answers
*Performance*	Yo-Yo Test	Number of completed shuttles
	Sargent test	hS1/hS2/hS3 jump height
*Perception/Motivation*	(Q1) Do you enjoy physical activity and sports?	Yes/No
	(Q2) Would you like to do it more often?	Yes/No
	(Q3) Do you play sports in the afternoon?	Yes/No
	(Q4) Do you enjoy participating in sporting competitions?	Yes/No
	(Q5) Are you satisfied with how high you jump and how fast you run?	Yes/No
	(Q6) Would you like to jump higher and run faster?	Yes/No
	(Q7) Would you like to train more with your teammates?	Yes/No
	(Q8) Would you like to play volleyball, basketball and football with your classmates?	Yes/No

**Table 2:** Descriptive Statistics.

Variable	M	SD	Shapiro–Wilk	*p*-Value
Height (m)	1.442	0.085	0.959	0.609
Weight (kg)	39.924	6.341	0.963	0.693
Yo-Yo Test (Shuttles)	4.235	1.033	0.876	0.028
Sargent Test hS1	21.588	5.328	0.949	0.446
Sargent Test hS2	24.529	4.274	0.963	0.685
Sargent Test hS3	25.941	3.766	0.940	0.323

M = mean; SD = standard deviation.

**Table 3:** Pearson’s Correlations Yo-Yo Test with Sargent Test.

Variable 1	Variable 2	Pearson’s r	*p*
Yo-Yo Test (Shuttles)	Sargent Test hS1	−0.106	0.685
Yo-Yo Test (Shuttles)	Sargent Test hS2	0.069	0.792
Yo-Yo Test (Shuttles)	Sargent Test hS3	−0.093	0.724

**Table 4:** Pearson’s Correlations with significant *p*-value.

Variable 1	Variable 2	Pearson’s r	*p*
Biological sex	Weight (Kg)	−0.562	0.019
Biological sex	Sargent Test hS1	−0.554	0.021
Biological sex	Sargent Test hS3	−0.499	0.041
Height (m)	Weight (Kg)	0.667	0.003
Weight (Kg)	Sargent Test hS3	0.544	0.024

**Table 5:** Descriptive frequencies of questionnaire responses by sex and statistically significant associations between questionnaire items identified by Chi-square analysis.

Questions	*MALE*		*FEMALE*		*Chi-Squared Analysis*	
	*Yes*	*No*	*Yes*	*No*	*Related Questions*	*p-Value*
*(Q1) Do you enjoy physical activity and sports?*	8	0	9	0	Q3	0.026
*(Q2) Would you like to do it more often?*	8	0	9	0	Q7	
*(Q3) Do you play sports in the afternoon?*	7	1	9	0	Q5	<0.001
*(Q4) Do you enjoy participating in sporting competitions?*	6	2	9	0	Q7	
*(Q5) Are you satisfied with how high you jump and how fast you run?*	7	1	7	2	Q6	0.001
*(Q6) Would you like to jump higher and run faster?*	8	0	7	2	Q7	
*(Q7) Would you like to train more with your teammates?*	7	1	7	2	Q8	0.003
*(Q8) Would you like to play volleyball, basketball and football with your classmates?*	6	2	6	3	Q7	
					Q3	0.005
					Q4	

Note. The frequencies reported for males and females are presented for descriptive purposes only. The *p*-values do not refer to comparisons between sexes. Instead, they represent the results of Chi-square tests examining the association between the questionnaire items listed in the “Related questions” column.

**Table 6:** Distribution and relationships between motor and perceptual variables.

Variable/Relationship	Statistical Test	*p*-Value
*Height*	Shapiro–Wilk	*p* > 0.05
*Weight*	Shapiro–Wilk	*p* > 0.05
*Sargent Test*	Shapiro–Wilk	*p* > 0.05
*Yo-Yo Test*	Shapiro–Wilk	*p* = 0.028
*Height—Weight*	Pearson’s correlation	*p* = 0.003
*Weight—Sargent Test hS3*	Pearson’s correlation	*p* = 0.024
***biological sex—Sargent Test hS3***	Pearson’s correlation	*p* = 0.041
*Yo-Yo Test—Sargent Test*	Pearson’s correlation	*p* > 0.05
*Afternoon sport practice—Training with teammates*	Chi-square	*p* < 0.05
*Training with teammates—Perceived motor competence*	Chi-square	*p* < 0.05
*Afternoon sport practice—Perceived motor competence*	Chi-square	*p* < 0.05

## Data Availability

The data presented in this study are available on request from the corresponding author. The data are not publicly available due to privacy.
